# Selective remodeling of snoRNA and sdRNA biology in glioblastoma

**DOI:** 10.3389/fcell.2026.1925311

**Published:** 2026-09-04

**Authors:** Hongtao Yin, Meiying Mu, Zhuoqun Wang

**Affiliations:** 1 Department of Neurology, Zibo Central Hospital, Zibo, Shandong, China; 2 Department of Oncology, Zibo Central Hospital, Zibo, Shandong, China

**Keywords:** extracellular vesicles, glioblastoma, liquid biopsy, ribosome biogenesis, small nucleolar RNA (snoRNA), snoRNA-derived RNA (sdRNA), therapy resistance, tumor metabolism

## Abstract

Small nucleolar RNAs (snoRNAs) are best known as guide RNAs for ribosomal RNA modification, but accumulating evidence indicates that their biology extends beyond canonical ribosome maturation. Across GBM-specific and broader glioma studies, snoRNAs, snoRNA-derived RNAs (sdRNAs), and associated small nucleolar ribonucleoprotein components are emerging as regulators of malignant cell states. This review frames GBM-associated snoRNA and sdRNA alterations as a cell-biological remodeling process that links ribosome biogenesis, metabolic adaptation, treatment response, and extracellular-vesicle output. Across currently available GBM and broader glioma studies, tumor-restraining C/D-box snoRNAs, including SNORD76, SNORD47, SNORD44, and SNORD113-3, tend to be reduced, and restoration of several of these molecules suppresses malignant phenotypes in their respective experimental systems. Conversely, tumor-supporting snoRNA-associated activities, including the U3–PHAX–DNA-PKcs–TRIM24 complex, a U3-derived small RNA acting through ZBTB7A, and preprint-based H/ACA snoRNA/snoRNP activity involving dyskerin, are maintained, increased, or functionally co-opted in specific GBM-related contexts. These alterations converge on three recurrent cellular contexts: translational capacity, glucose and glycolipid metabolism, and survival under radiotherapy or temozolomide. Treatment-associated senescence may also reshape extracellular-vesicle snoRNA cargo, with SNORA49 detected in a small longitudinal plasma series, although this remains exploratory rather than a validated liquid-biopsy marker. We also emphasize the need to distinguish snoRNAs and sdRNAs from their SNHG host transcripts, because these molecular entities have distinct biogenesis and mechanisms. Together, these studies suggest a context-dependent pattern of snoRNA and sdRNA dysregulation across GBM and related glioma models, with the strength of evidence varying among individual molecular axes. SnoRNAs and sdRNAs should be viewed as an emerging regulatory layer in GBM rather than as established therapeutic targets. Future work should validate molecule-specific snoRNA and sdRNA axes in disease-relevant models and determine whether extracellular-vesicle snoRNAs provide reproducible readouts of treatment-associated cell states.

## 1 Introduction

Small nucleolar RNAs (snoRNAs) have one of the most settled job descriptions in molecular biology. Primarily localized to the nucleolus, they guide 2′-O-methylation and pseudouridylation during ribosomal RNA maturation, thereby fine-tuning ribosome assembly. Some snoRNAs are also processed into smaller fragments, termed snoRNA-derived RNAs (sdRNAs), which have historically received less attention (Wang Y. et al., 2025; Ye et al., 2025; Jouravleva and Zamore, 2025). For decades, this canonical housekeeping role was regarded as the principal function of snoRNAs, while their relevance to cancer remained comparatively underexplored (Wang Y. et al., 2025; Ye et al., 2025; Jouravleva and Zamore, 2025). Meanwhile, microRNAs and long non-coding RNAs became established regulators of glioma proliferation, invasion, and therapeutic resistance (Kovalenko et al., 2021; Majed et al., 2026), and recent glioma-focused work has placed circular RNAs (circRNAs) within the same landscape through microRNA sequestration, RNA-binding-protein interactions, transcriptional control, and, in selected cases, peptide translation (Wang H. et al., 2025).

CircRNA dysregulation has been linked to DNA-damage responses, programmed cell death, angiogenesis, metabolic reprogramming, tumor-microenvironment communication, and temozolomide resistance (Wang H. et al., 2025). Against this comparatively mature literature, snoRNAs and sdRNAs remain less systematically integrated into glioma biology, partly because host transcripts, mature snoRNAs, and processed sdRNAs are easily conflated. This view is now changing. Across multiple tumor types, snoRNAs and sdRNAs have been found to be dysregulated and functionally consequential (Wang Y. et al., 2025; Ye et al., 2025; Jouravleva and Zamore, 2025). This raises a central question for glioblastoma: is its altered snoRNA biology merely an accompaniment to malignancy, or does the tumor actively exploit it?

Studies in glioma and GBM suggest a bidirectional pattern in which tumor-suppressive C/D-box snoRNAs are downregulated (Xia et al., 2020; Chen et al., 2015; Xu et al., 2017; Cui et al., 2025), whereas oncogenic snoRNAs, their ribonucleoprotein partners, and sdRNAs are retained or co-opted (Xu et al., 2024; Dong et al., 2023; Guardia et al., 2025). These alterations converge on ribosome remodeling, metabolic rewiring, and survival under therapy (Xia et al., 2020; Chen et al., 2015; Xu et al., 2017; Cui et al., 2025; Xu et al., 2024; Dong et al., 2023; Guardia et al., 2025; Yokogami et al., 2022), while part of this program may also be reflected in circulating extracellular vesicle cargo (DeLuca et al., 2026). Here, “selective remodeling” is used as a descriptive, hypothesis-generating framework rather than as evidence of evolutionary selection or a coordinated tumor-wide program. It denotes the recurrent directional pattern reported across independent studies: reduction of tumor-restraining snoRNAs together with retention, elevation, or noncanonical use of tumor-supporting snoRNA or snoRNP activities. Current evidence does not establish that these alterations co-occur within individual tumors or arise from a common regulator, and further molecule-resolved profiling and functional validation will be required to distinguish this pattern from alternative explanations such as nucleolar stress or differences in cellular composition. Throughout this review, “GBM,” “glioma,” “lower-grade glioma,” and “GBM-like” are used according to the original disease or model context, with cross-context findings explicitly identified as extrapolations.

## 2 snoRNAs, sdRNAs, and SNHG transcripts: distinct molecular entities

A snoRNA is a short non-coding RNA, roughly sixty to three hundred nucleotides long, that guides the chemical modification of ribosomal RNA. Most snoRNAs localize to the nucleolus, whereas a related subset, the small Cajal-body-specific RNAs (scaRNAs), localize to Cajal bodies and guide the modification of spliceosomal snRNAs (Wang Y. et al., 2025; Ye et al., 2025; Jouravleva and Zamore, 2025). Two families are defined by their conserved sequence motifs. C/D box snoRNAs, working with fibrillarin and its partner proteins, direct 2′-O-methylation; H/ACA box snoRNAs, built around dyskerin, direct pseudouridylation. In each case the snoRNA base-pairs with its target and the assembled small nucleolar ribonucleoprotein (snoRNP) installs the modification at a precise position, tuning the ribosome as it is built. In vertebrates, many snoRNAs are not transcribed as independent units but are excised from the introns of host genes. This is the canonical role, and for a long time it was assumed to be the whole story (Wang Y. et al., 2025; Ye et al., 2025; Jouravleva and Zamore, 2025).

However, the canonical role does not encompass the full biological repertoire of snoRNAs. A subset of snoRNAs is further processed into shorter, metabolically stable fragments termed snoRNA-derived RNAs (sdRNAs) through either Dicer-dependent or Dicer-independent pathways (Ender et al., 2008; Taft et al., 2009; Godang et al., 2023). These fragments generally fall within the small-RNA size range of approximately 18–30 nucleotides, with many H/ACA-derived species clustering around 20–24 nucleotides (Taft et al., 2009). Some sdRNAs associate with Argonaute-containing RNA-induced silencing complexes and repress target messenger RNAs through microRNA-like mechanisms (Ender et al., 2008; Taft et al., 2009; Coley et al., 2022a). Several sequences initially annotated as miRNAs were subsequently recognized as snoRNA-derived fragments (Scott and Ono, 2011; Wajahat et al., 2021; Coley et al., 2022b). Evidence from multiple animal systems and human cancers therefore supports *bona fide* regulatory functions for at least a subset of sdRNAs (Ender et al., 2008; Taft et al., 2009; Godang et al., 2023; Coley et al., 2022a; Scott and Ono, 2011; Wajahat et al., 2021; Coley et al., 2022b; Bratkovič et al., 2020). However, not every detected snoRNA fragment is necessarily functional, and current approaches do not always distinguish a regulatory sdRNA from a processing by-product.

**FIGURE 1:**
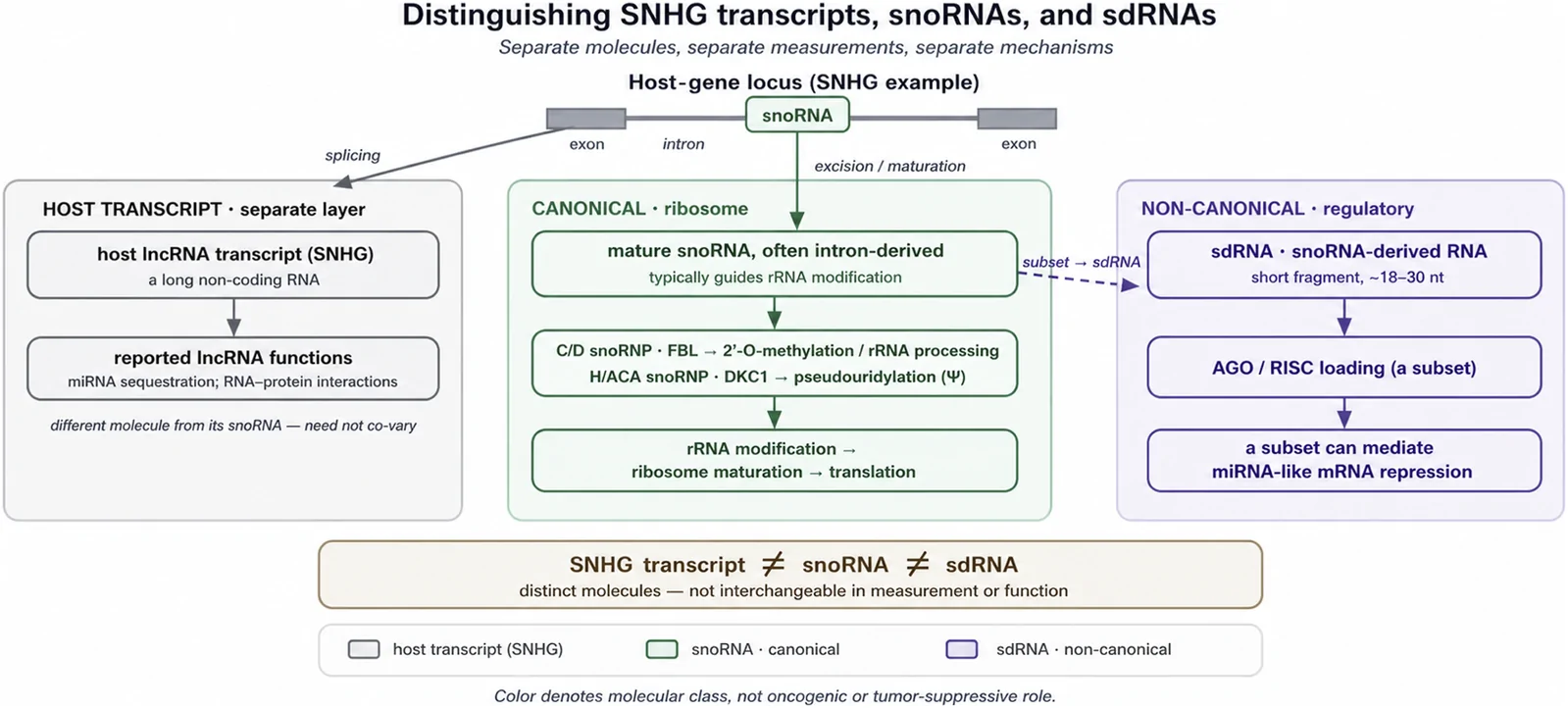
Distinguishing SNHG transcripts, snoRNAs, and sdRNAs as separate molecular entities. A host-gene transcript may contain an intronic snoRNA, but the host transcript, the mature snoRNA, and any snoRNA-derived RNA (sdRNA) are distinct molecules with different biogenesis and potential mechanisms. Mature snoRNAs participate in canonical ribonucleoprotein complexes, including C/D-box snoRNPs associated with fibrillarin and 2′-O-methylation, and H/ACA snoRNPs associated with dyskerin and pseudouridylation. A subset of snoRNAs can be further processed into sdRNAs, some of which may associate with AGO/RISC and mediate microRNA-like repression. The SNHG host transcript is shown separately because its expression or function should not be inferred from detection of the embedded snoRNA or sdRNA. Color denotes molecular class rather than oncogenic or tumor-suppressive role.

A further source of ambiguity is the relationship between snoRNAs and their host transcripts. Many snoRNAs are encoded within the introns of longer transcripts. Small nucleolar RNA host gene (SNHG) transcripts constitute a prominent class of long non-coding RNAs and can have functions independent of the snoRNAs they harbor. Some SNHG transcripts, for example, have been reported to act as competing endogenous RNAs by sequestering microRNAs (Yu et al., 2025; Wu, 2023; Chu et al., 2021). The host transcript and its embedded snoRNA are distinct molecular species with different biogenesis and mechanisms, and their expression need not change in parallel (Yu et al., 2025; Wu, 2023; Chu et al., 2021). Accordingly, detection of a snoRNA does not establish the abundance or function of its host transcript. Without molecule-specific measurements and perturbations, an observed phenotype cannot be confidently attributed to the SNHG transcript, the embedded snoRNA, or an sdRNA derived from it. Distinguishing these entities is therefore essential for interpreting snoRNA-associated changes in glioma. The relationships among SNHG transcripts, snoRNAs, and sdRNAs are summarized in Figure 1. These associative, biomarker-oriented, and selected functional findings are summarized in Table 1.

**TABLE 1:** Associative, biomarker-oriented, and selected functional snoRNA evidence in glioma.

snoRNA/signature	Evidence type	Glioma context	Direction/finding	Strength	Source
7-snoRNA signature	Prognostic signature	Lower-grade glioma	Independent predictor of survival; correlates with immune infiltration	*Associative*	Zhou et al. (2023)
SNORD88C	Function (within 7-snoRNA panel)	Glioma cells	Promotes proliferation, invasion, migration	Functional in glioma cell lines; not GBM-specific or clinically validated	Zhou et al. (2023)
11-snoRNA signature	Prognostic signature	Lower-grade glioma	Independent predictor; immune-infiltration differences	*Associative*	Deng et al. (2021)
5 methylated snoRNA genes	Methylation signature	Glioma	Independent prognostic predictor	*Associative*	Yue et al. (2022)
SNORA71B	Function (within 5-gene panel)	Glioma cells	Silencing restrains proliferation/migration; lowers mesenchymal and cell-cycle markers	Functional in glioma cell lines; not GBM-specific or clinically validated	Yue et al. (2022)
SNORA12	Prognostic + immune	Combined TCGA GBM/LGG cohort; osteosarcoma and NK-cell experiments	Prognostic/immune associations in GBM/LGG; TIGIT regulation demonstrated only in non-glioma systems	Extrapolated; not GBM-validated	He et al. (2026)
snoRNA loci (methylome)	Immune association	PD-L1–high vs. PD-L1–low GBM	Methylation enriched at regulatory-RNA/snoRNA loci	*Associative*	Hutarew et al. (2022)
snoRNA EV cargo (incl. SNORA49)	Extracellular vesicle (bioRxiv preprint)	Patient-derived GBM cultures; paired unenriched plasma EVs (n = 4)	snoRNA enrichment in 4/5 culture models; SNORA49 increased after treatment in 3/4 patients	Exploratory; preprint; plasma signal origin unresolved	DeLuca et al. (2026)
EV liquid-biopsy platform	Technology context	Plasma from mixed glioma subtypes, including GBM	Supports EV mRNA genotyping feasibility; does not validate a snoRNA biomarker	Technology context only	Song et al. (2025)

Most entries are correlative; the few with direct functional support are marked Functional, and the exploratory nature of the extracellular-vesicle signal is marked in the Strength column. SNORA12’s TIGIT link is from osteosarcoma rather than glioma and is flagged as extrapolated. “Functional” indicates direct perturbation in the specified experimental model and does not imply clinical validation; evidence strength is assigned to individual claims rather than to the multigene panel as a whole.

## 3 Beyond rRNA guidance: opposing snoRNA activities in glioma and GBM

**FIGURE 2:**
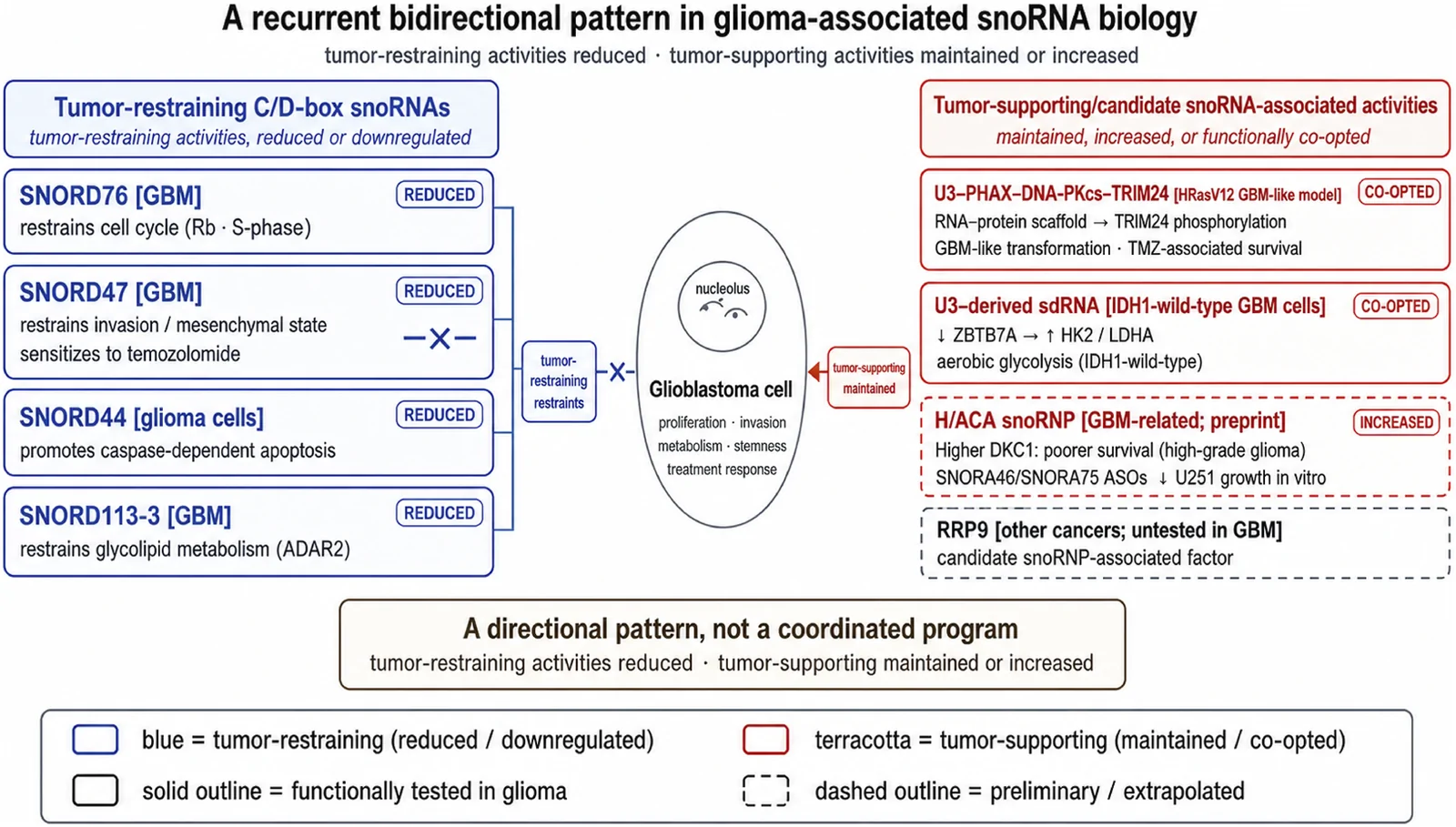
Recurrent bidirectional pattern in glioma-associated snoRNA biology. Tumor-restraining C/D-box snoRNAs, including SNORD76, SNORD47, SNORD44, and SNORD113-3, tend to be reduced in glioma or GBM, whereas tumor-supporting snoRNA-associated activities are retained, increased, or functionally co-opted in specific contexts. These include the U3–PHAX–DNA-PKcs–TRIM24 complex, the U3-derived small RNA acting through ZBTB7A, and H/ACA snoRNA/snoRNP activity involving dyskerin. RRP9 is included as a potential extension from other cancers and has not yet been tested in GBM. Color denotes proposed functional direction; solid outlines indicate functionally tested evidence, whereas dashed outlines indicate preliminary, associative, or extrapolated evidence. Disease and model contexts are indicated beside the corresponding nodes. The figure summarizes a recurrent directional pattern rather than a coordinated snoRNA program.

Available studies suggest that glioma-associated snoRNA alterations occur in opposing functional directions across heterogeneous disease and model contexts. On the oncogenic side, U3 provides the most mechanistically detailed GBM-related example, acting as a structural scaffold in an HRasV12/TRIM24-engineered epithelioid-GBM-like model (Xu et al., 2024). The U3-associated protein RRP9 has also shown oncogenic activity in several other cancers and is included in Figure 2 as a potential extension that remains to be tested in GBM (Du et al., 2021; Zhang et al., 2022; Li et al., 2025). On the suppressive side, SNORD76 and SNORD47 have direct GBM-model support, whereas SNORD44 and SNORD113-3 have been studied in broader glioma or GBM-relevant systems (Xia et al., 2020; Chen et al., 2015; Xu et al., 2017; Cui et al., 2025). Across these studies, tumor-restraining snoRNAs tend to be reduced, whereas tumor-supporting snoRNA or snoRNP activities tend to be retained, increased, or repurposed (Figure 2). This recurrent directional pattern motivates the selective-remodeling framework, while its broader coordination across individual GBM tumors remains to be established.

## 4 Prognostic snoRNA signatures and a therapy-associated extracellular vesicle signal

Transcriptomic and methylation-based studies have identified snoRNA signatures associated with prognosis and immune features in glioma. Seven- and eleven-snoRNA expression signatures stratified patients with lower-grade glioma, whereas a five-methylated-snoRNA-gene signature was developed in broader glioma cohorts (Zhou et al., 2023; Deng et al., 2021; Yue et al., 2022). Functional follow-up of individual candidates further showed that SNORD88C promotes proliferation, invasion, and migration, whereas SNORA71B silencing suppresses these phenotypes and reduces mesenchymal and cell-cycle markers (Zhou et al., 2023; Yue et al., 2022). The seven- and eleven-snoRNA signatures were also associated with immune-cell infiltration and checkpoint-related features (Zhou et al., 2023; Deng et al., 2021). In a combined TCGA GBM–lower-grade glioma cohort, SNORA12 expression was associated with prognosis and immune features, while its proposed relationship with TIGIT was functionally examined in osteosarcoma and NK-cell systems (He et al., 2026). Differential methylation of regulatory RNA loci, including snoRNAs, has also been reported between PD-L1-high and PD-L1-low GBM samples (Hutarew et al., 2022). Together, these studies support the prognostic and biological relevance of selected snoRNAs, although functional validation remains limited to a subset of individual candidates.

Treatment-associated changes in extracellular-vesicle snoRNAs may provide an additional layer of information. A 2026 bioRxiv preprint reported that radiation-induced senescence may reshape the extracellular snoRNA landscape of GBM (DeLuca et al., 2026). In patient-derived GBM cultures, extracellular vesicles released after irradiation-induced senescence showed snoRNA enrichment in four of five models, accompanied by enrichment of several snoRNA-associated proteins (DeLuca et al., 2026). In paired plasma samples from four patients with GBM, SNORA49 increased after treatment in three cases (DeLuca et al., 2026). These findings suggest that extracellular-vesicle snoRNAs may provide a circulating readout of treatment-associated senescence, although larger and independently validated studies are required (DeLuca et al., 2026). More broadly, extracellular-vesicle-based molecular profiling supports the feasibility of monitoring glioma-associated molecular alterations through vesicular nucleic acids (Song et al., 2025).

## 5 snoRNA alterations in translation, metabolism, and treatment response

**FIGURE 3:**
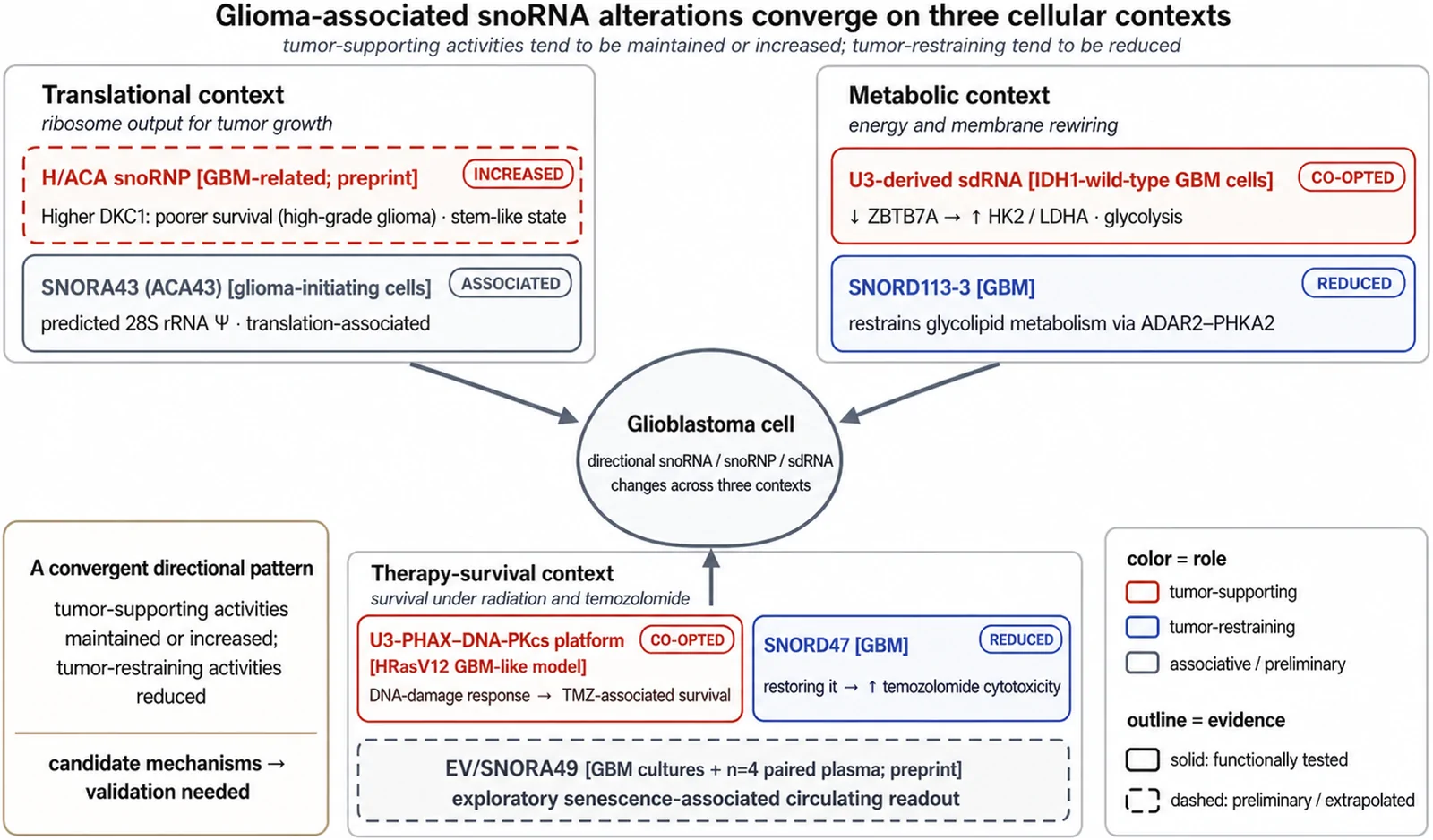
Convergence of glioma-associated snoRNA alterations on translation, metabolism, and treatment response. Reported snoRNA, snoRNP, and sdRNA alterations can be organized around three recurrent cellular contexts: translational capacity, metabolic adaptation, and survival under therapy. In the translation context, H/ACA snoRNA/snoRNP activity and SNORA43 (ACA43) connect snoRNA biology with ribosome-associated and stem-like glioma states. In the metabolic context, a U3-derived small RNA supports glycolysis through ZBTB7A, whereas SNORD113-3 restrains glycolipid metabolism through the ADAR2–PHKA2 axis. In the treatment-response context, the U3–PHAX–DNA-PKcs platform and SNORD47 are linked to opposing effects on temozolomide-related survival, while senescence-associated extracellular-vesicle snoRNA cargo, including SNORA49, is shown as an exploratory circulating readout. Terracotta indicates tumor-supporting activities, blue indicates tumor-restraining activities, and gray denotes associations whose directional role in GBM remains uncertain. Disease/model context and evidence status, including preprint-derived findings, are indicated beside the corresponding nodes.

Across GBM-specific studies, broader glioma datasets, and GBM-like experimental models, the reported snoRNA alterations can be organized into three recurring cellular contexts: translational capacity, metabolic adaptation, and survival under therapy (Figure 3).

Ribosome biogenesis provides the most immediate connection between canonical snoRNA function and glioma biology. A 2025 Research Square preprint that had not undergone journal peer review at the time of this revision reported higher expression of several H/ACA snoRNAs and snoRNP components, including dyskerin (DKC1), in GBM-associated datasets and models than in normal cortex and lower-grade glioma (Guardia et al., 2025). Its survival analysis combined grade III glioma and GBM, the functional growth experiment used antisense-mediated depletion of SNORA46 and SNORA75 in U251 cells, and separate neuronal-differentiation analyses included non-GBM systems (Guardia et al., 2025). These results support an association between H/ACA activity and proliferative or poorly differentiated states, but they neither establish a GBM-specific survival effect nor demonstrate that altered pseudouridylation causally maintains GBM (Guardia et al., 2025). Metabolic state may intersect with the same ribosomal axis. In patient-derived glioma-initiating cultures, methionine deprivation reduced SNORA43 (reported as ACA43), an H/ACA snoRNA predicted to guide pseudouridylation of 28S rRNA, together with large-ribosomal-subunit gene expression and overall translation (Yokogami et al., 2022). Methionine deprivation also altered S-adenosylmethionine availability, cholesterol synthesis, and stemness-associated programs, so the study does not isolate SNORA43/ACA43 as the sole mediator (Yokogami et al., 2022). Pharmacological inhibition of cholesterol synthesis likewise lowered SNORA43/ACA43 and stemness markers, consistent with a cholesterol–rRNA axis acting upstream of this snoRNA (Yokogami et al., 2022). Together, these findings connect H/ACA snoRNA biology with translational capacity and stem-like states in glioma models, but a causal chain from specific pseudouridylation changes to GBM maintenance remains to be established.

Metabolic rewiring in glioma is linked to snoRNA-derived regulators that act in opposing directions. In IDH1-wild-type GBM cells, a U3-derived small RNA reduces ZBTB7A mRNA stability and protein abundance, thereby relieving ZBTB7A-mediated repression of HK2 and LDHA and sustaining aerobic glycolysis (Dong et al., 2023). Conversely, SNORD113-3 is downregulated in GBM. Its re-expression promotes ADAR2 dimerization and stability, increases A-to-I editing of PHKA2 mRNA, and consequently suppresses PHKA2-dependent glycolipid metabolism (Cui et al., 2025). Together, these findings connect snoRNA biology with glucose and glycolipid metabolism in glioma. A similar directional pattern is seen in metabolism: the glycolysis-supporting U3-derived fragment is functionally active in IDH1-wild-type GBM cells, whereas the glycolipid-restraining SNORD113-3 is downregulated.

Responses to radiotherapy and temozolomide provide a third context in which snoRNA-associated mechanisms may influence cellular survival under treatment. In an HRasV12/TRIM24-engineered epithelioid GBM-like transformation model, U3 snoRNA recruits Ku-dependent DNA-PKcs, while activated PHAX recruits TRIM24 to the U3-containing complex (Xu et al., 2024). This mechanism was demonstrated in an engineered GBM-like system rather than in a conventional patient-derived GBM model. Conversely, re-expression of the downregulated tumor-suppressive SNORD47 increased temozolomide cytotoxicity in GBM cells (Xu et al., 2017). Radiation also induces senescence in GBM cells and non-neoplastic brain cells. In preclinical models, senescence-associated secretory factors released by irradiated brain cells can promote glioma regrowth (Fletcher-Sananikone et al., 2021; Dehghan et al., 2024). A bioRxiv preprint reported that extracellular vesicles from irradiated, senescent patient-derived GBM cultures were enriched in snoRNAs, while plasma-EV SNORA49 increased after treatment in three of four patients (DeLuca et al., 2026). These findings suggest that extracellular-vesicle snoRNAs may provide a circulating readout of treatment-associated senescence.

## 6 Discussion and limitations

The evidence reviewed here supports a cautious but coherent interpretation: snoRNA biology in glioma is not limited to housekeeping ribosome maturation. Several snoRNAs, snoRNP components, and snoRNA-derived fragments have been linked to tumor growth, metabolism, treatment response, and extracellular-vesicle cargo (Xia et al., 2020; Chen et al., 2015; Xu et al., 2017; Cui et al., 2025; Xu et al., 2024; Dong et al., 2023; Guardia et al., 2025; Yokogami et al., 2022; DeLuca et al., 2026). Across the available examples, tumor-supporting activities tend to be retained or increased, whereas tumor-restraining snoRNAs tend to be reduced. This directional pattern provides a useful framework for organizing the field, but it should not be read as proof of a single coordinated snoRNA program in GBM.

This review has several limitations. The number of molecule-specific functional axes directly tested in GBM-relevant systems is still limited. SNORD76, SNORD47, SNORD113-3, and the U3-derived RNA–ZBTB7A axis have GBM-specific or GBM-relevant experimental support, whereas SNORD44 has been studied in broader glioma material and established U87/U251 models, and the U3–PHAX–DNA-PKcs–TRIM24 pathway was demonstrated in an HRasV12/TRIM24-engineered epithelioid GBM-like model (Xia et al., 2020; Chen et al., 2015; Xu et al., 2017; Cui et al., 2025; Xu et al., 2024; Dong et al., 2023). Beyond these mechanistic examples, much of the available evidence remains associative, including multigene prognostic signatures, methylation models, immune associations, and the relationship between DKC1 expression and survival (Guardia et al., 2025; Zhou et al., 2023; Deng et al., 2021; Yue et al., 2022; He et al., 2026; Hutarew et al., 2022). Evidence for sdRNA function is even more limited in GBM, as much of the broader mechanistic framework derives from non-glioma systems; within GBM, the U3-derived RNA–ZBTB7A axis remains one of the few directly characterized examples (Dong et al., 2023; Ender et al., 2008; Taft et al., 2009; Godang et al., 2023; Coley et al., 2022a; Scott and Ono, 2011; Wajahat et al., 2021; Coley et al., 2022b; Bratkovič et al., 2020).

The SNORA12–TIGIT relationship was originally identified in osteosarcoma and NK-cell systems, providing a potentially relevant mechanistic clue for GBM. However, the brain tumor microenvironment differs substantially from peripheral tumors in its immune composition, neural and glial cellular context, and blood–brain barrier characteristics (Sarkaria et al., 2018; Klemm et al., 2020). Whether this relationship also operates in GBM therefore remains an important question for future studies using disease-relevant models.

Small-RNA sequencing and bulk transcriptomic analyses may not reliably distinguish mature snoRNAs from derived fragments, or embedded snoRNAs from their SNHG host transcripts (Jouravleva and Zamore, 2025; Taft et al., 2009; Bratkovič et al., 2020; Yu et al., 2025; Wu, 2023; Chu et al., 2021). This creates a risk of misattributing a phenotype to the wrong molecular species. Future work should therefore combine molecule-specific detection, precise perturbation, and rescue strategies. Disease-relevant systems are also essential, including patient-derived glioma stem-like cells, orthotopic models, therapy-stressed cultures, and, where feasible, single-cell or single-vesicle approaches.

The translational implications are promising but early. Restoring tumor-suppressive snoRNAs such as SNORD47, inhibiting tumor-supporting snoRNA-associated pathways, or monitoring treatment-associated extracellular-vesicle snoRNA cargo are plausible directions, but none is yet clinically validated (Xu et al., 2017; Xu et al., 2024; Guardia et al., 2025; DeLuca et al., 2026). Delivery to intracranial tumors, selectivity over normal ribosome biology, biomarker reproducibility, and cohort size remain major barriers. Locoregional approaches, including convection-enhanced delivery and intracavitary depots, can bypass the blood–brain barrier and increase drug exposure at the resection margin (Narsinh et al., 2024). Systemic nanocarriers provide a less invasive alternative; notably, a phase 0 study of BCL2L12 siRNA spherical nucleic acids demonstrated intratumoral accumulation and target engagement in recurrent GBM (Kumthekar et al., 2021). Focused ultrasound can also transiently increase blood–brain barrier permeability and may offer another route to enhance intracranial delivery (Narsinh et al., 2024; Sonabend et al., 2023). Together, these strategies illustrate the expanding toolkit for RNA delivery in neuro-oncology, although effective snoRNA-directed therapy will ultimately require sufficient tumor coverage, cellular uptake and endosomal escape, as well as selectivity for tumor cells over normal neural and ribosome-biogenesis programs. The extracellular-vesicle signal is particularly attractive because GBM is difficult to sample repeatedly, but current patient data are too limited to define a validated liquid-biopsy marker (DeLuca et al., 2026).

Overall, snoRNAs and sdRNAs should be viewed as an emerging regulatory layer in GBM rather than as established therapeutic targets. Their value at present lies in clarifying candidate dependencies, separating functional mechanisms from associations, and identifying molecular readouts that merit further testing. The central next step is not broader speculation, but rigorous validation of specific snoRNA and sdRNA axes in models that reflect the biology and treatment context of GBM.
